# Sonication fluid culture of antibiotic-loaded bone cement spacer has high accuracy to confirm eradication of infection before reimplantation of new prostheses

**DOI:** 10.1186/s13018-021-02520-4

**Published:** 2021-06-13

**Authors:** Qingyu Zhang, Baocong Ding, Jinglin Wu, Jun Dong, Fanxiao Liu

**Affiliations:** 1 Department of Orthopedics, Shandong Provincial Hospital affiliated to Shandong First Medical University, No.324, Road Jing Wu Wei Qi, Jinan, 250021 Shandong China; 2 Rehabilitation Department, Shandong University of Traditional Chinese Medicine Affiliated Hospital, No.16369, Road Jing Shi, Jinan, 250014 Shandong China; 3 Basic Course Department, Weihai Vocational College, New Sci-Tech Park of Beihai, Weihai, 264200 Shandong China

**Keywords:** Periprosthetic joint infection, Sonication culture, Spacer, Meta-analysis

## Abstract

**Background:**

Sonication fluid culture of antibiotic-loaded bone cement spacer has been used to predict reinfection of two-stage revision, but its value remains disputable. This study aims to evaluate the association between the culture result of the sonicated spacer and the status of patients with periprosthetic joint infection receiving two-stage revision.

**Materials and methods:**

A comprehensive electronic literature search was performed through four databases including PubMed, Embase/Ovid, and EBSCO, and the Cochrane Library to retrieve studies in which sonication fluid culture of the antibiotic spacer was conducted before reimplantation. The pooled sensitivity, specificity, positive likelihood ratio (PLR), negative likelihood ratio (NLR), and diagnostic odds ratio (DOR) were calculated to assess the association between the culture result of sonicated spacer and prognosis of the two-stage revision.

**Results:**

Eleven eligible studies comprising 603 artificial joints with PJI (134 suffering a clinical failure of two-stage revision) were included in the quantitative analysis. The pooled incidences of positive culture of sonicated spacer and intraoperative tissue were 0.14 (95% confidence interval [CI] 0.08–0.21) and 0.14 (95% CI 0.08–0.20), respectively. A positive culture of sonicated antibiotic-loaded bone cement spacer illustrated moderate sensitivity (0.31, 95% CI 0.13–0.58) but high specificity (0.94, 95% CI 0.86–0.98) for the diagnosis of therapeutic failure of two-stage revision; the pooled DOR was 7.67 (95% CI, 3.63–16.22). Meanwhile, the pooled sensitivity, specificity, and DOR of intraoperative tissue culture during the two-stage revision to predict therapeutic failure were 0.32 (95% CI, 0.20–0.47), 0.96 (95% CI, 0.92–0.98), and 10.62 (95% CI, 4.90–23.01), respectively.

**Conclusions:**

Sonication fluid culture of antibiotic-loaded bone cement spacer revealed high accuracy for confirming eradication of infection before reimplantation of new prostheses and therefore could be used as a supplement for assessing therapeutic effect for PJI. However, both sonication fluid culture and intraoperative tissue culture from antibiotic-loaded bone cement spacer showed restricted yield for the prediction of a septic failure after the two-stage revision of PJI. Large-scale, prospective studies are still needed to testify current findings.

## Introduction

**Fig. 1:**
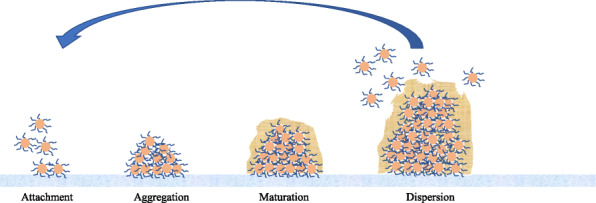
Steps of microorganism biofilm formation and development

Two-stage exchange arthroplasty is the most commonly recommended treatment protocol for chronic periprosthetic joint infection (PJI) of the knee and hip arthroplasty [1]. After removal of the infected implants and debridement of infected and necrotic tissue, an articulating or static bone cement (polymethyl methacrylate/PMMA) spacer impregnated with antimicrobial agents is inserted to preserve the joint space and enable local antibiotic delivery; then, a systemic antibiotic therapy was administrated before a second prosthesis [2]. Although high success rate had been reported with an infection eradication rate above 80% for both total knee [3] and hip [4] arthroplasties, there are still risks of revision failure involving persistent or recurrent infection after reimplantation [5, 6]. Presumably being multi-factorial, the formation of biofilm-forming pathogens (Fig. 1) on the bone cement spacer is likely the paramount cause for the infection to persist [5, 6]. Once the embedded antibacterial molecules have exhausted, the antibiotic-loaded bone cement spacer (ACS) itself can serve as an optimal biomaterial surface to which bacteria can adhere, grow, and develop antibiotic resistance [7]. Meanwhile, relapse of PJI may be more frequent when drug-resistant organisms such as methicillin-resistance *Staphylococcus aureus* (MRSA) and methicillin-resistant Coagulase-negative Staphylococci (CoNS) were involved [2, 8].

Failure to eradicate PJI at the second-stage surgical procedure is associated with a decreased chance of favorable prognosis with a subsequent staged revision [9, 10] and may predispose patients with this catastrophic complication to the amputation, knee fusion, or Girdlestone procedure, leading to joint dysfunction and limited joint mobility [5, 11]. Hence, an accurate method to predict persistent/recurrent infection after two-stage revision is highly desirable to avoid placing a new prosthesis in an infected environment. Unfortunately, conventional tools such as systemic inflammatory biomarkers, white blood cell count, the culture of synovial fluid, and intraoperative histopathology all revealed low ability to assess the infection eradication and treatment failure [8, 12, 13]. A recent study performed by our team revealed that plasma fibrinogen was an excellent biomarker for diagnosing PJI, comparable to serum CRP and ESR, while the diagnostic value of circulating D-dimer was only moderate [14]. However, given the fact that the biofilm formed on the prosthesis surface may prevent microorganisms from being detected and eliminated, the culture of the sonicated prosthesis explanted during primary revision has been demonstrated to be more sensitive than the conventional periprosthetic tissue culture for diagnosing PJI [15, 16]. Inspired by these findings, a series of small studies supported the application of sonicated culture of removed spacers before reimplantation and illustrated an association between a positive result, persistent or recurrent infection, and poor outcome [17–19]. However, these studies only included a small number of participants using qualitative, semi-quantitative, and quantitative sonication spacer fluid culture, making these results hard to be widely generalized. Moreover, data from other authors reported different experiences with the value of sonication fluid culture of bone cement spacer in comparison with conventional intraoperative tissue culture in the management of recurrent and persistent PJI [20, 21].

In order to further clarify this issue, a systematic literature search was conducted to retrieve studies evaluating the association between culture result of sonicated antibiotic-loaded bone cement spacers and occurrence of therapeutic failure after a two-stage revision procedure. Moreover, a meta-analysis was performed to reach more comprehensive results and provide high-quality evidence for the decision-making of clinicians.

## Materials and methods

The methodological approach to evidence searching and data synthesis described in the current study and meta-analysis was in line with the standards of the Preferred Reporting Items for a Systematic Review and Meta-analysis of Diagnostic Test Accuracy Studies (PRISMA-DTA) [22]. No ethical approval or informed consent was required in this article because all data were retrieved from published literature. Study searching, eligibility identification, data extraction, and quality assessment were performed by two investigators independently. Any disagreement would have to be resolved through discussion, and the two researchers would have to reach a consensus.

### Search strategy

Four electronic databases (PubMed, Embase/Ovid, and EBSCO, and the Cochrane Library) were searched for entries recorded from the time of database inception to March 15, 2021, using a combination of keywords and MeSH terms including “spacer” and “periprosthetic joint infection”. No limitations were imposed on the journal and language of publication. Meanwhile, bibliographies of relevant articles were also hand-screened to retrieve any additional possible records.

### Inclusion criteria

Studies included in systematic review need to meet all following criteria: (1) participants, patients receiving two-stage revision for PJI; (2) intervention, sonication fluid culture of removed spacers; (3) control, the diagnosis of PJI was confirmed by the Musculoskeletal Infection Society (MSIS), American Academy of Orthopaedic Surgeons (AAOS), or Infectious Diseases Society of America (IDSA) guidelines; (4) outcome, adequate data could be extracted to calculate the positive incidence of sonication fluid culture or diagnostic accuracy of this test for persistent infection; and (5) study design, diagnostic accuracy study.

Exclusion criteria were (1) case reports/series, meta-analyses, editorials, commentaries, expert opinion, and narrative reviews, and (2) studies in which PJI could not be diagnosed with golden standards.

The titles and abstracts were independently assessed in an unblinded standardized manner for eligibility. The final decision regarding eligibility was based on the full-article scrutinizing. If more than one study provided overlapping data, only the most comprehensive or latest one was included.

### Data extraction and quality assessment

Requisite data extracted and recorded to standardized excel files included surname of the first author, year and region of publication, study inclusion interval, study design, demographic characteristics of enrolled participants (e.g., sex, age), criteria for the diagnosis of PJI, the method to conduct culture of sonication spacer, number of positive/negative case of sonication fluid culture, and prognosis of two-stage revision. Clinical failure was defined as recurrence/existence of PJI after two-stage revision.

The methodological quality of included studies was appraised according to the QUADAS (Quality Assessment of Diagnostic Accuracy Studies)-2 tool which contains four key domains, namely, patient selection, index test, reference standard, and flow and timing [23]. The risk of bias in each domain and concerns about applicability were assessed in the first three domains. Questions answered with “yes” indicated a low risk of bias/concern, “no” a high risk of bias/concern, and “unclear” that relevant information was not provided [23].

### Statistical analyses

The pooled positive incidence of sonication fluid culture of the explanted spacer was computed by generating the proportion of the yield (true-positive) and associated 95% confidence intervals (CI) using a random effects model. For the diagnostic modalities, true-positive (TP), false-positive (FP), true-negative (TN), and false-negative (FN) results were extracted from the two-by-two contingency table to calculate the pooled sensitivity, specificity, positive likelihood ratio (PLR), negative likelihood ratio (NLR), diagnostic odds ratio (DOR), and summary receiver operating characteristic (sROC)**.** Heterogeneity among the included studies was assessed using the *I*^2^ statistic. An *I*^2^ value of 0–50% implied non-significant heterogeneity, and values of > 50% indicated substantial heterogeneity. Publication bias was performed using Deeks’ funnel plot asymmetry test. All meta-analyses were conducted using the STATA (V. 12.0, StataCorp, College Station, TX). The value of a two-sided *p* < 0.05 was considered statistically significant in all statistical tests.

## Results

### Selection process

**Fig. 2:**
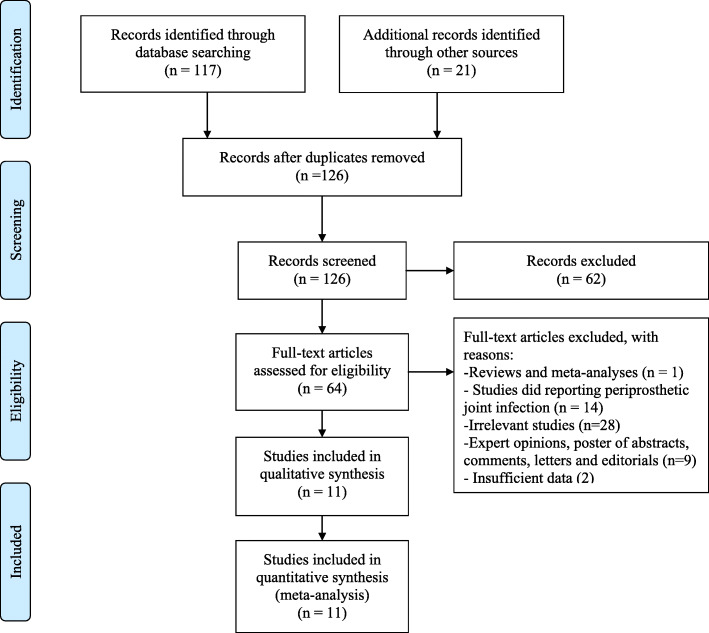
A flow diagram of the selection process of the included studies

A total of 117 potentially eligible articles were retrieved after the original search of three electronic databases, and 21 additional articles were retrieved from the references of relevant articles (meta-analyses, systematic reviews, letters, editorials, and guidelines). We excluded 62 ineligible articles by screening the titles and abstracts. After reading the full text of the remaining articles, 11 studies [17, 19–21, 24–30] involving a total of 583 participants were included in the statistical analysis, all being published in English. The study selection process is presented as a flow chart in Fig. 2.

### Study characteristics

The detailed characteristics of the enrolled studies are summarized in Table 1. These studies were published from 2012 to 2019 with the sample sizes ranged from 13 to 157. A total of 583 participants involving 603 artificial joints were included, among which therapeutic failure (persistent or recurrent infection) occurred in 134 joints. The included studies investigated artificial hip, knee, shoulder, and elbow joints. Three studies [17, 20, 26] are conducted in the USA, and eight [19, 21, 24, 26–30] from Europe. Ten studies [17, 19, 20, 24–30] provided the results of sonication fluid culture from spacers, and one [21] used PCR analysis of sonication fluid from spacers. Nine studies [17, 19, 24–30] also provided the culture results of intraoperative tissue of bone cement spacers. Five [17, 19, 21, 28, 29] studies used a prospective design and the others [20, 24–27, 30] a retrospective cohort design. The QUASDAS-2 scores of qualities ranged from 9 to 11 with a mean of 10.27. Treatment failure was defined as existence of infection after two-stage revision for PJI.

**Table 1:** Basic information of the included studies

Study	Country	No. of patients	Design	Inclusion interval	Sex (M/F)	Mean age (range, yrs)	No. of joints					Reference standard	No. infected or failure/total No. of prostheses	DM (n, %)	Tobacco use (n, %)	COPD (n, %)	Corticosteroid use (n, %)	Immuno-suppression	Rheumatoid arthritis	QUADAS-2 score
							Hips	Knees	Shoulders	Elbows	Others									
Sorli et al. [19]	Spain	55	P	01.2007–09.2008	24/31	73 (39–98)	17	37	1	0	0	PUS, SIGNS, and M	18/55	14 (25)	-	6 (11)	6 (11)	-	-	11
Mariconda et al. [30]	Italy	21	R	03.2009–1.2011	11/10	66.1 (55–78)	5	16	0	0	0	SIGNS, and M, ECRP, ELC	3/21	-	-	-	-	-	-	10
Nelson et al. [17]	USA	36	P	01.11.2010–30.11.2011	19/17	68 (20–86)	7	29	0	0	0	RI, SS, CAS, AMP, and DEA	11/36	12 (33)	11 (31)	-	11 (31)	2 (6)	-	11
Bereza et al. [28]	Poland	13	P	NR	6/7	69.2 (50–84)	4	9	0	0	0	M and/or SS	2/13	-	-	-	-	-	-	9
Esteban et al. [29]	Spain	46	P	10.2010–12.2013	11/35	71.3 (NR)	13	32	2	3	0	F or P with ECRP or GIT	16/50	-	-	-	-	-	-	11
Mariaux et al. [21]	Switzerland	30	P	09.2014–03.2016	22/8	66 (28–85)	15	14	0	0	1	M; SIGNS;F	6/46	-	-	-	-	-	-	11
Olsen et al. [20]	USA	41	R	09.2013–04.2016	21/20	62 ± 11	15	25	0	0	1	MSIS	6/41	8 (20)	9 (22)	-	-	-	-	10
Torrens et al. [27]	Spain	21	R	01.2006–12.2014	7/14	67.5 (45–86)	0	0	21	0	0	M, PUS; SIGNS;SS	3/21	4 (18)	-	-	2 (8)	-	-	10
Gomez-Urena et al. [26]	USA	66	R	11.2007–10.2014	42/24	60.5 (51–71)	20	46	0	0	0	PUS; sinus tract; H; SIGNS with ECRP or EESR	15/66	12 (18)	-	-	-	7 (11)	5 (8)	10
Hipfl et al. 24]	Germany	97	R	06.2014–11.2016	41/56	69.7 ± 11.2	0	97	0	0	0	SY; ELC; GIT; H	22/97	21 (22)	-	-	10 (10)	-	-	10
Sambri et al. [25]	Italy	157	R	06.2014–07.2016	64/93	63 (21–93)	55	102	0	0	0	MSIS	32/157	-	-	-	-	-	-	10

*R* retrospective; *P* prospective; *NR* not recorded; *DM* diabetes mellitus; *COPD* chronic obstructive pulmonary disease; *Delphi* Delphi-based consensus data; *P* pain; *M* microbiology; *SS* subsequent surgery; *H* histopathology; *GIT* grossly infected tissues; *RI* recurrent infection; *CAS* chronic antibiotic suppression; *AMP* amputation; *DEA* death; *PUS* pus was found in the synovial fluid, implant site, or sinus tract; *SIGNS* clinical signs of acute inflammation; *MSIS* Musculoskeletal Infection Society; *F* fistulae; *ECRP* elevated C-reactive protein; *EESR* elevated erythrocyte sedimentation rate; *ELC* elevated leukocyte count; *SY* symptoms or signs of prosthetic joint infection

### Qualitative analysis

#### Incidence of a positive result

**Fig. 3:**
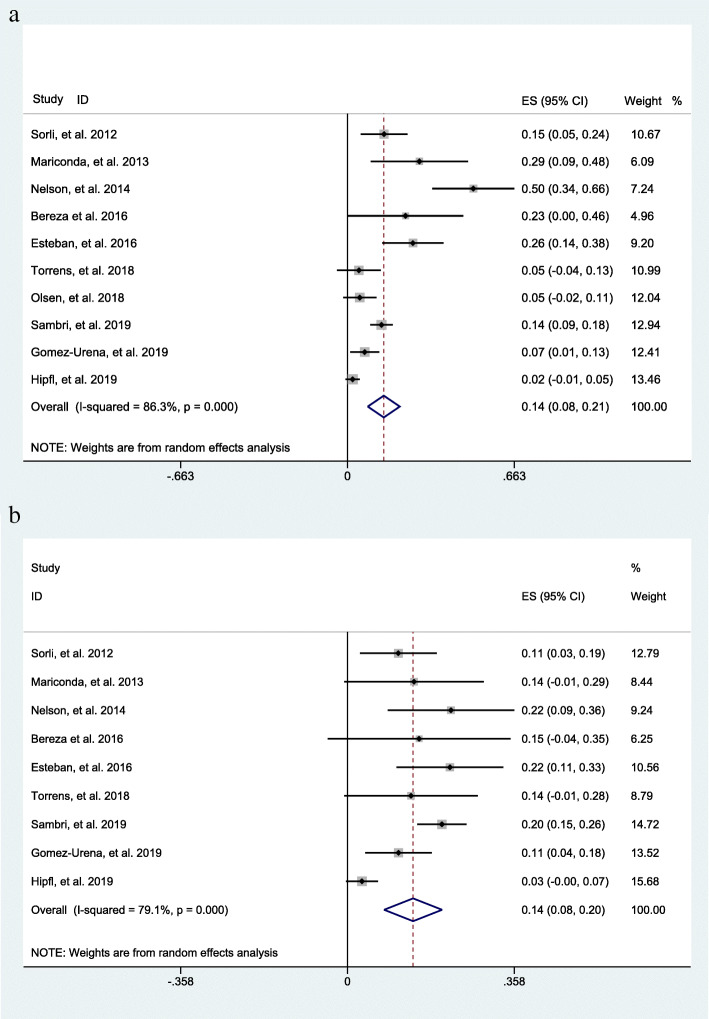
**a** The overall incidence of a positive result of sonication fluid culture of bone cement spacers in patients receiving two-stage exchange revision. **b** The overall incidence of a positive result of the intraoperative tissue culture of bone cement spacers in patients receiving two-stage exchange revision

The overall incidence of a positive result of sonication fluid culture of bone cement spacers as generated from 10 datasets [17, 19, 20, 24–30] was 14% (95% CI 12–21%) at the second surgical stage during a two-stage exchange procedure (Fig. 3a). For the intraoperative tissue culture of bone cement spacers, the overall incidence as generated from 9 datasets [17, 19, 24–30] was 14% (95% CI 8–20%), demonstrating a similarly positive result with sonication fluid culture (Fig. 3b).

#### Diagnostic accuracy of sonicated spacer culture

**Fig. 4:**
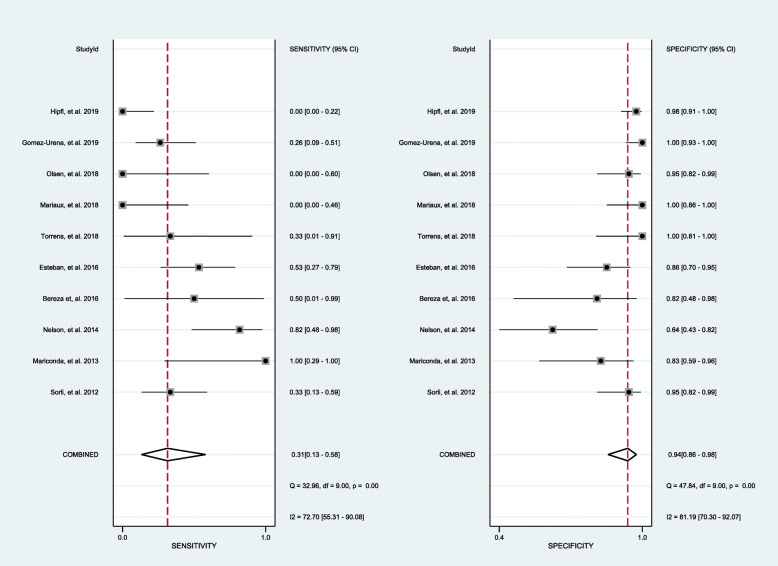
The pooled sensitivity and specificity of sonication fluid culture of bone cement spacers in patients receiving two-stage exchange revision

**Fig. 5:**
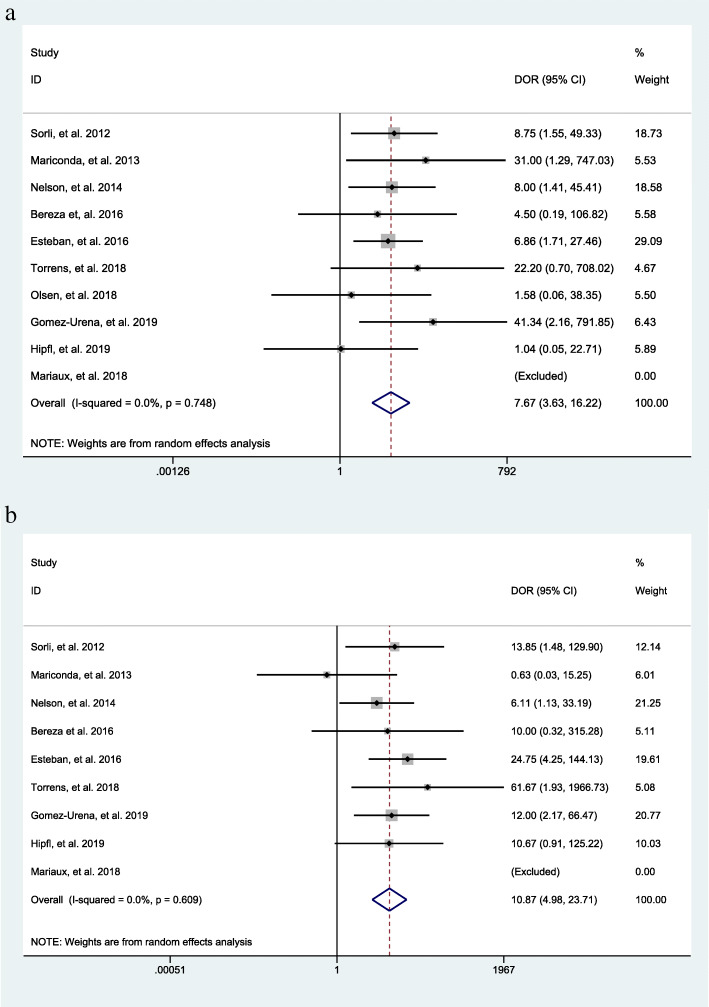
The diagnostic odds ratio of sonication fluid culture (**a**) or intraoperative tissue culture (**b**) of bone cement spacers in patients receiving two-stage exchange revision

**Fig. 6:**
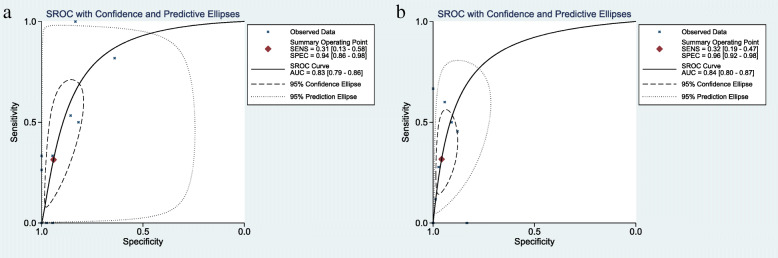
The systemic receiver operating curve of sonication fluid culture (**a**) or intraoperative tissue culture (**b**) of bone cement spacers in patients receiving two-stage exchange revision

**Fig. 7:**
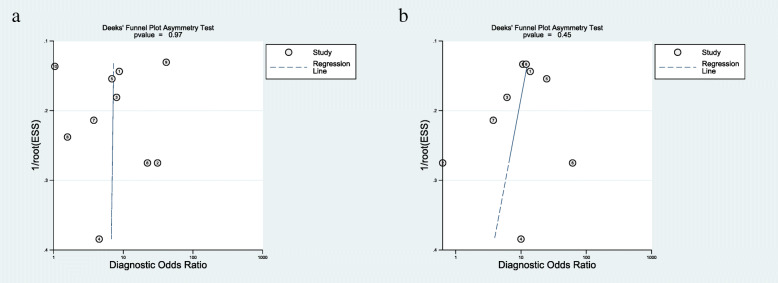
Graphical display of the results of Deek’s test for publication bias of sonication fluid culture (**a**) and intraoperative tissue culture (**b**)

Results assessing the value of sonication fluid culture of antibiotic-loaded bone cement spacer for diagnosing revision failure as generated from the 10 datasets [17, 19–21, 24, 26–30] included in the present meta-analysis showed a sensitivity of 0.31 (95% CI, 0.13 to 0.58), a specificity of 0.94 (95% CI, 0.86 to 0.98), a PLR of 5.37 (95% CI, 2.83 to 10.20), an NLR of 0.73 (95% CI, 0.53 to 0.99), a DOR of 7.38 (95% CI, 3.33 to 16.38), and an AUC of 0.83 (95% CI, 0.79 to 0.86) (Figs. 4, 5a, and 6a). The threshold effect was found in the provided data (Spearman correlation coefficient = 0.753; *p* value = 0.007). Deeks’ funnel plot asymmetry test revealed no publication bias (*p* value = 0.97) (Fig. 7a).

#### Diagnostic accuracy of intraoperative tissue culture

**Fig. 8:**
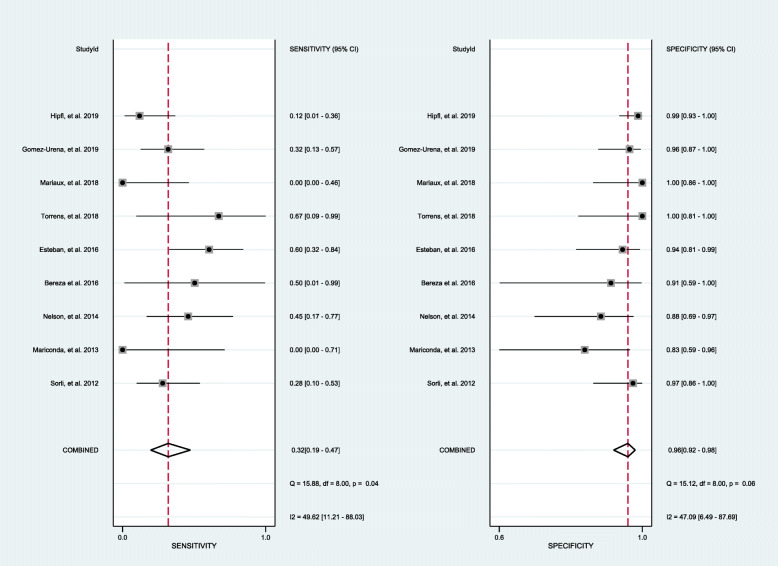
The pooled sensitivity and specificity of intraoperative tissue culture of bone cement spacers in patients receiving two-stage exchange revision

Performance assessing the diagnostic value of tissue culture of antibiotic-loaded bone cement spacer for PJI as generated from the 9 datasets [17, 19, 21, 24, 26–30] included in the present meta-analysis showed a sensitivity of 0.32 (95% CI, 0.20 to 0.47), a specificity of 0.96 (95% CI, 0.92 to 0.98), a PLR of 7.58 (95% CI, 3.86 to 14.85), an NLR of 0.71 (95% CI, 0.59 to 0.87), a DOR of 10.62 (95% CI, 4.90 to 23.01), and an AUC of 0.84 (95% CI, 0.80 to 0.87) (Figs. 5b and 6b, 8). The threshold effect was not found in the provided lesion-based data (Spearman correlation coefficient= 0.345; *p* value = 0.328). Deeks’ funnel plot asymmetry test revealed no publication bias (*p* value = 0.45) (Fig. 7b).

## Discussion

The two-stage exchange is the standardized protocol in the treatment of PJI as it not only provides local antimicrobial delivery to the joint but also preserves the normal anatomy of the joint space, facilitating delayed reimplantation. However, there are still cases of revision failure where the cement spacer itself may act as a biomaterial surface that predisposes the survival and proliferation of microorganisms [7]. Moreover, the early identification of the pathogens involved in septic failure after reimplantation may help in giving pathogen-specific suppressive antibiotic therapy as soon as possible after surgery [31]. The prediction of a persistent/recurrent infection is mainly achieved by the culture of intraoperative tissue or synovial fluid before replacement of new implants, and various synovial or serological biomarkers, but a definitive diagnosis of this catastrophic complication remains a tough issue [32, 33]. The current study demonstrated that the sonication fluid culture of explanted spacers revealed a similar incidence (14%) of a positive result to that of intraoperative tissue culture. These two methods showed comparable diagnostic sensitivity (31% and 32%, respectively) and specificity (94% and 96%, respectively) for the prediction of septic failure of two-stage revision.

Septic failure of a two-stage revision of PJI can be attributed to either insufficient debridement of the previous infection (persistent infection) or a newly onset infection with a different pathogen [34]. These two groups of patients pose distinct predisposing factors and infection causes [8]. A previous study involving 37 patients with PJI caused by oxacillin-resistant staphylococci reported that septic failure of two-stage revision occurred in 24.32% (9/37) cases, despite all patients having negative periprosthetic tissue cultures at the time of reimplantation of new prostheses, from which 44.44% (4/9) of patients had a recurrent infection with the same organism and 55.56% (5/9) revealed reinfection with a different organism [8]. In Hipfl et al.’ study [24], 22 patients receiving two-stage revisions of total knee arthroplasty suffered clinical failure, and only 3 reinfections are caused by the same organism. In Olsen et al.’s study [20], two cases with positive sonication fluid culture of spacers revealed distinct results with the initial infection. Although isolation of the same pathogen from periprosthetic tissue culture and ACS culture served as the golden diagnostic criteria of persistent PJI, since genotypic analysis on bacterial strains was not conducted in included studies, these cases considered as relapses might be new infections not detected during the second surgery or that occurred after implantation. Several recent studies indicated that septic failure after two-stage revisions was mainly due to the new infection, rather than the persistent infection [13, 35]. The ACS, as a foreign body, could not only allow persistence of preexisting infection but also facilitate adherence of new organism. It had to be emphasized that the complicated and prolonged procedures of two-stage exchange were indeed associated with a higher risk of introducing new pathogens in comparison with the primary procedure [36]. Other possible explanations for these newly identified pathogens were that these patients had a polymicrobial infection, and the additional species were not revealed at the first-stage surgery or only represent a contamination of the sample [24]. These reasons could partially explain why microbiological tests before reimplantation of new artificial joints, such as intraoperative tissue culture and sonication fluid culture of the explanted prostheses and inflammatory markers, lack sensitivity to detect subsequent infections during the second-stage surgery [35]. Therefore, further studies are needed to seek host factors, genetic predisposition, and misconduct of medical staff as causes of septic failure of staged revision of PJI.

In our study, although a significant association between results of sonication fluid culture of the antibiotic-loaded spacer and aseptic failure was identified (DOR = 7.38; 95% CI, 3.33 to 16.38), positive results of culture were only identified in less than one-third of patients with septic failures. Meanwhile, there were discordant results between periprosthetic tissue culture and sonication fluid culture of spacers. In Sambri et al.’s study [25], concordant results between two culture methods occurred in 186 (83.8%) out of 222 cases. Among 31 cases with persistent infection and discordant results of two culture methods, 23 were only identified according to the periprosthetic tissue cultures of spacers and 8 only from cultures performed on sonication fluids of spacers. In addition to a new infection, the great majority of false-negative cases can be explained by the fact that the spacer would release antimicrobials out of the cement and result in inhibition of bacterial growth in sonication fluid culture [21, 37]. Mariaux et al. reported an antimicrobial concentration at or above minimal inhibitory concentration (MIC) of common PJI microorganisms in sonication fluid [21]. Meanwhile, preoperative antibiotic treatment can also affect the sonication fluid culture of bone cement spacer. Trampuz et al. reported that the sensitivities of periprosthetic tissue and sonication fluid cultures for diagnosing periprosthetic joint infection were 60.8% and 78.5%, respectively in patients without antimicrobial therapy more than 14 days before surgery, and declined to 45.0% and 75.0% in those with an antibiotic course within 14 days before surgery [33]. Various antibiotic-free period before the second-stage surgical procedure may also influence the sensitivity of sonication fluid culture of bone cement spacer for the diagnosis of septic failure of the two-stage exchange protocol for PJI.

Albeit the sensitivity of ACS culture is limited, the utmost caution must be exercised when pathogens are isolated from sonication fluid of the cement spacer, particularly a highly resistant microorganism or same bacteria that caused the initial PJI. Long-term elution of antibiotics from bone cement spacer may lead to drug resistance in bacterial strains [38]. Before reimplantation of the prosthesis during a two-stage exchange protocol, a sonication fluid culture of the bone-cement spacer should be conducted to provide more information for the screening of infection. Meanwhile, different perspectives exist concerning the therapeutic regimen of different versus identical pathogens when comparing culture results of sonication fluid with primary PJI. In fact, for a patient with positive sonication fluid culture of bone cement spacer, an applicable algorithm should be established on host characteristics, organism species/virulence, timing, and other variables.

There are limitations that should be considered with regard to the interpretation of current findings. The first is the lack of a standardized reference test for the diagnosis of septic failure of two-stage infection. Another minor limitation is that during the merging of diagnostic data, subgroup analyses on the basis of important information such as amount of culture fluid and duration of follow-up were not conducted due to the limited number of studies. Only 11 studies were included in this meta-analysis, and most (6/11) of them were retrospective in nature. Third, the sample size of these studies was quite small, and hence, this meta-analysis might be subject to variability and inadequacy in data collection. Lastly, yet importantly, evidence of heterogeneity existed throughout the included studies, which was a significant limitation of the current investigation. Therefore, we emphasized the pooled incidence, and DOR, which is a global measure encompassing other diagnostic parameters as the main outcome of interest to reflect the performance of sonication fluid culture of bone cement spacer.

## Conclusions

In conclusion, sonication fluid culture of antibiotic-loaded bone cement spacer revealed high accuracy for confirming eradication of infection before reimplantation of new prostheses and therefore could be used as a supplement for assessing therapeutic effect for PJI. However, both sonication fluid culture and intraoperative tissue culture from antibiotic-loaded bone cement spacer showed restricted yield for the prediction of a septic failure after the two-stage revision of PJI. Large-scale, prospective studies are still needed to testify current findings.

## Data Availability

All data analyzed during this study are included in this published article.

## Abbreviations

AAOS: American Academy of Orthopaedic Surgeons
ACS: Antibiotic-loaded bone cement spacer
CI: Confidence intervals
CoNS: Coagulase-negative Staphylococci
DOR: Diagnostic odds ratio
FN: False negative
FP: False positive
IDSA: Infectious Diseases Society of America
MIC: Minimal inhibitory concentration
MRSA: Methicillin-resistance *Staphylococcus aureus*
MSIS: Musculoskeletal Infection Society
NLR: Negative likelihood ratio
PJI: Periprosthetic joint infection
PLR: Positive likelihood ratio
PMMA: Polymethyl methacrylate
PRISMA-DTA: Preferred Reporting Items for a Systematic Review and Meta-analysis of Diagnostic Test Accuracy Studies
QUADAS: Quality assessment of diagnostic accuracy studies
sROC: Summary receiver operating characteristic
TN: True negative
TP: True positive
